# Overexpression of miR-4433 by suberoylanilide hydroxamic acid suppresses growth of CML cells and induces apoptosis through targeting Bcr-Abl

**DOI:** 10.7150/jca.34972

**Published:** 2019-09-07

**Authors:** Haiyan Wu, Jingyi Yin, Zhengdong Ai, Guiming Li, Yan Li, Li Chen

**Affiliations:** 1 Department of Pathophysiology, Medical School, Kunming University of Science and Technology, Kunming, Yunnan 650500, China;; 2 Department of Cadre Health, The First Affiliated Hospital of Yunnan Province, Kunming, Yunnan, China;

**Keywords:** chronic myelogenous leukemia, Bcr-Abl, SAHA, miR-4433, growth, apoptosis

## Abstract

**Background**: Targeting Bcr-Abl is the key for the treatment of CML. Although great progress has been achieved for the treatment of CML patients in chronic stage, effective drugs with good safety are not available for those in advanced stages of CML patients. In present study, a histone deacetylase inhibitor, suberoylanilide hydroxamic acid (SAHA), was used to screen for microRNA that can target Bcr-Abl.

**Methods**: RT-qPCR was used to determine Bcr-Abl and miR-4433 transcription level in CML cells. In CML cells, Proteins including PARP, caspase-3, acetyl-histone 3, histone 3 and Bcr-Abl, as well as Bcr-Abl downstream proteins were detected using western blot. Cell viability and apoptosis were monitored respectively by MTS assay and flow cytometry. The correlation between miR-4433 and Bcr-Abl was determined by luciferase reporter assay. The anti-tumor effect of miR-4433 to K562 cells was evaluated by nude mouse xenograft model in vivo.

**Results**: SAHA up-regulated the acetylation level of histone 3, and effectively inhibited Bcr-Abl mRNA level and its downstream signal transduction pathway, while inhibiting the growth of CML cells and inducing apoptosis. Furthermore, bioinformatics tools predicted that miR-4433 is a putative microRNA targeting Bcr-Abl and that the expression level of miR-4433 was significantly increased after SAHA treatment in K562 cells. Luciferase activity analysis revealed that miR-4433 directly targets Bcr-Abl. Additionally, transient expression of miR-4433 abrogated Bcr-Abl activity and its downstream signaling pathways while inducing apoptosis in K562 cells. Moreover, stable expression of miR-4433 suppressed Bcr-Abl and its downstream signaling pathway, and inhibited the growth of K562 cells in vitro and the growth of K562-xenografts in nude mice.

**Conclusion**: miR-4433 was identified as a microRNA targeting Bcr-Abl, which may be subject to epigenetic regulation of SAHA, a histone deacetylase inhibitor that has been approved by the US FDA for the treatment of cutaneous T-cell lymphoma. The findings of this study provide a molecular basis from another angle for the use of SAHA in the treatment of CML.

## Introduction

Chronic myelogenous leukemia (CML) is a myeloproliferative disorder characterized by the presence of Bcr-Abl which is the product of a fusion gene formed by a reciprocal chromosomal translocation t (9; 22)(q34;q11) 1, 2. It is estimated that Bcr-Abl is found in approximately 90% of newly diagnosed patients with CML and 30% patients with acute lymphoblastic leukemia 1-3. Bcr-Abl harbors aberrant tyrosine kinase activity and exerts its oncogenic function by activating a cascade of intracellular signaling pathways including Ras-Raf-mitogen-activated protein (MAP) kinase, Janus kinase/signal transducer and activators of transcription (Jak-Stat), and phosphatidylinositol-3 (PI3) kinase/Akt, which ultimately leads to increased cell proliferation and survival, and escape from apoptosis 4. Thus, targeting Bcr-Abl has been an important strategy for CML treatment 5, 6. Imatinib mesylate (STI571, Gleevec, Novartis), a first-generation tyrosine kinase inhibitor, effectively inhibits the Bcr-Abl activity by occupying the ATP-binding pocket of Bcr-Abl, thus abrogating subsequent signal transduction pathways 2. It is estimated that nearly 82% newly diagnosed chronic phase patients with CML showed complete cytogenetic response on treatment with imatinib over a median follow-up of 54 months 7. However, resistance to imatinib develops over the time 6, 8, and to overcome the acquired resistance, second and third generation tyrosine kinase inhibitors (e.g., dasatinib, nilotinib and ponatinib) have been developed. Despite great progress has been achieved by these tyrosine kinase inhibitors in controlling chronic phase CML, effective drugs with good safety are not available for those CML patients in advanced stages. Therefore, development of novel chemotherapeutics with low toxicity and high efficiency for patients in advanced stages of CML is urgently needed.

microRNAs (miRNAs) are small non-coding RNA molecules (18-25 nt in length) that modulate the gene expression at post-transcriptional level in multicellular organisms by complementary interaction with the 3'-untranslated regions (3'-UTR) of target mRNA 9. Previous studies have reported that microRNAs are regulated by epigenetic mechanisms, and up-regulation of microRNAs by epigenetic drugs can lead to down-regulation of target oncogenes 10, 11. Epigenetic regulation of gene and/or microRNA expression is primarily mediated by DNA methylation and histone acetylation 12. Histone deacetylation is known to be correlated with transcriptional silencing and down-regulation of pro-apoptotic gene expression, especially in cancer cells 13. The histone deacetylase inhibitors (HDACIs) suppress the activity of histone deacetylases (HDACs), and epigenetically modulate the gene expression, including those of genes associated with cell cycle arrest and apoptosis 14.

Previous studies reported that epigenetically up-regulated microRNAs by DNA methylase inhibitor or HDACIs presented oncogene-targeted functions and inhibited cell proliferation in some kinds of tumors, including CML 15, 16. In present study, suberoylanilide hydroxamic acid (SAHA), a histone deacetylase inhibitor, was used to screen for microRNAs which can target Bcr-Abl, and a microRNA miR-4433 was identified. Furthermore, the anti-tumor activities of miR-4433 in CML cell K562 were investigated.

## Materials and Methods

### Chemicals and antibodies

**Figure 1:**
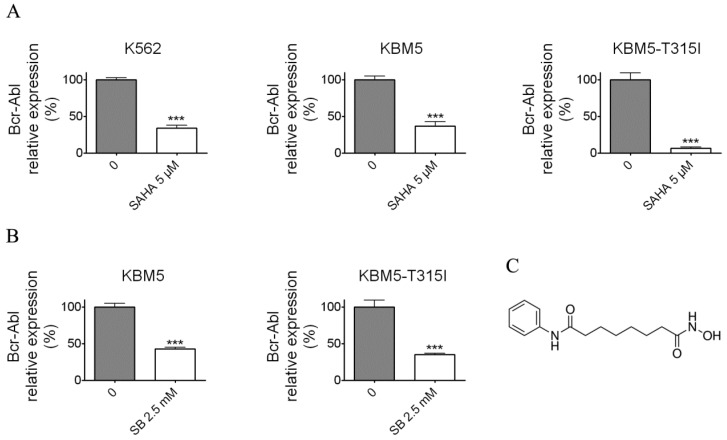
Histone deacetylase inhibitors (SAHA and Sodium butyrate (SB)) reduced the Bcr-Abl mRNA levels in CML cell lines. (A) K562 expressing wild-type Bcr-Abl, imatinib-sensitive KBM5 and imatinib-resistant KBM5-T315I CML cells were treated with SAHA (5 μM) for 48 hours and the Bcr-Abl mRNA levels were determined by qRT-PCR. (B) KBM5 and KBM5-T315I cells were treated with SB (2.5 mM) for 48 hours and the Bcr-Abl mRNA levels were examined. (C) The molecular structure of suberoylanilide hydroxamic acid (SAHA). ***, *p* < 0.001, Student's *t* test.

SAHA (suberoylanilide hydroxamic acid, Fig. 1C, purity > 98%, HPLC, SML0061) 17, sodium butyrate (SB, B5887) were purchased from Sigma (Shanghai, China). SAHA was dissolved in DMSO (Sigma, Shanghai) at a stock concentration of 20 mM, and stored at -20°C. Antibodies against poly (adenosine diphosphate [ADP]-ribose) polymerase (PARP), phospho-c-Abl atY245, c-Abl, phospho-Erk1/2 (T202/Y204), Erk1/2, phospho-Akt at Ser473, Akt, Caspase-3, Acetyl-Histone 3 (Lys9, C5B11), Histone 3 (96C10) were bought from Cell Signaling Technologies (Shanghai, China). Antibodies against phospho-STAT5A/B (Tyr694/699) and STAT5A/B were obtained from Millipore (Shanghai, China). Goat anti-mouse poly-HRP and goat anti-rabbit poly-HRP were purchased from Thermo Scientific (Shanghai, China).

### Cell culture

CML cells K562 were grown in RPMI 1640 (Invitrogen, Shanghai, China) supplemented with 10% heat-inactivated fetal bovine serum. Imatinib-sensitive CML cells KBM5 expressing wild-type Bcr-Abl were cultured in Iscove's modified Dulbecco's medium (Invitrogen, Shanghai, China) supplemented with 10% fetal bovine serum. Imatinib-resistant CML cells KBM5-T315I bearing a substitution of threonine-to-isoleucine at 315 codon were maintained in the same medium as KBM5 but with 1.0 μM imatinib, which was removed before experiments with a wash-out periods of 2-3 days 18. Cells in logarithmic phase were used in all experiments starting with 2 × 10^5^ cells/ml.

### Cell viability assay

Cell viability was evaluated by MTS assay (CellTiter 96 Aqueous One Solution Cell Proliferation assay; Promega, Madison, WI) as previous described 18. 100 μl cells (2× 10^5^ cells/ml) were seeded in 96-well plates and incubated with various concentrations of SAHA for 72 hours. Four hours prior to culture termination, 20 μl MTS solution was added to each well. Absorbance was read on a 96-well plate reader at a wavelength of 490 nm. The drug concentration resulting 50% inhibition of cell growth (IC_50_) was calculated.

### Western blotting

Western blotting was performed using standard methods as previously described 18. Whole cell lysates were prepared in radio-immunoprecipitation assay buffer (1× PBS, 1% NP40, 0.5% sodium deoxycholate, 0.1% SDS) supplemented with freshly added 10 mM β-glycerophosphate, 1 mM orthovanadate, 10 mM NaF, 1 mM phenylmethylsulfonyl fluoride, and 1 × Roche Complete Mini Protease Inhibitor Cocktail. The DNA in the lysates was sheared by sonication with eight 1-second bursts at medium power. Cellular proteins were separated on 10-15% SDS-PAGE.

### Transfection

The miR-4433 duplexes mimics and negative control (NC) were synthesized by GenePharma (Shanghai, China). miR-4433 mimics sequence was 5'-ACAGGAGUGGGGGUGGGACAU-3' (duplexes). NC was siRNA duplexes (5'-UUCUCCGAACGUGUCACGUTT-3') with non-specific sequences. The transfections were performed using Lipofectamine 2000 (Invitrogen, Shanghai) according to the manufacturer's protocol. The final concentration of miRNA or siRNA was 100 nM. Forty eight hours post-transfection, cells were harvested for the real-time qPCR, western blot and flow cytometry analysis.

### Real-time qPCR

Total cellular RNA was extracted from cells by using the Trizol reagent (Invitrogen, Shanghai, China). For the Bcr-Abl expression, total RNA was reverse transcribed into cDNA (MMLV reverse transcriptase, Promega, Beijing), the level of gene expressions were measured by GoTaq qPCR Master Mix (Promega, Beijing) using ABI7000 cycler (Applied Biosystems, USA). The miRNA expression analysis were performed by use of miRcute miRNA first-strand cDNA synthesis kit (Tiangen Biotech, Beijing) and miRcute miRNA qPCR detection kit (Tiangen Biotech, Beijing) according to the manufacturer's protocol. The primers for real-time quantitative PCR were as follows: Bcr-Abl: forward primer 5'-TCCACTCAGCCACTGGATTTAA-3', reverse primer 5'-TGAGGCTCAAAGTCAGATGCTACT-3'; 18S: forward primer 5'-AAACGGCTACCACATCCAAG-3', reverse primer 5'-CCTCCAATGGATCCTCGTTA-3'; miR-4433: forward primer 5'- ACAGGAGTGGGGGTGGGAC -3', reverse primer 5'-GGCCACGCGTCGACTAGTAC-3'. PCR was performed at 94°C for 5 min and then 94°C for 30 s and 60°C for 30 s for 40 cycles. Relative quantification of gene or miRNA expression was performed by using the threshold cycle difference method, and the geometric mean of 18S or U6 level was used as an internal control to normalize the variability in expression level.

### Flow cytometry analysis

Cells were harvested, washed with PBS and subjected to Annexin V-FITC/propidium iodide (PI) double staining (Annexin V-FITC Apoptosis Detection Kit, Sigma Aldrich, Shanghai, China) according to the manufacturer's instruction. The number of viable, necrotic and apoptotic cells were counted by use of flow cytometer (Becton Dickinson, USA).

### Luciferase assay

miR-4433 putative binding sites of ABL1 (3'UTR) was amplified by PCR from complementary DNA (cDNA) of K562 cells using the PrimeSTAR Max DNA Polymerase (Takara, Beijing). Mutant miR-4433 putative binding sequence of ABL1 (3'UTR) was generated by overlap-extension PCR method. The two sequences were inserted downstream of the renilla luciferase coding region between Xho I and Not I restriction enzyme sites of the psiCHECK2 (Promega, Beijing), and the constructed plasmids were nominated as psiCHECK2-ABL1-UTR and psiCHECK2-ABL1-UTRm. The transfection were carried out with the psiCHECK2, psiCHECK2-ABL1-UTR and psiCHECK2-ABL1-UTRm after 293T cells reached 50% confluence, and 6 hours later the miR-4433 and NC duplexes were transfected. Firefly and renilla luciferase activities were determined forty eight hours post transfection using Dual-Luciferase Reporter Assay System (Promega, Beijing) on a microplate reader (FLX800, BioTech, US). The relative luciferase activity was calculated by normalizing the renilla luciferase activity to firefly luciferase activity. See the primer sequences for PCR in Supplementary Table 1.

### K562 cells stable expressing miR-4433 (K562-miR-4433)

Two synthesized oligonucleotide sequence (Supplementary Table 1) annealed cassette for miR-4433 expression were inserted into pSIH1-H1-Puro shRNA Expression Lentivector from System Biosciences (Shanghai, China). Lenti-viruses carrying miR-4433 were packaged by transfection of three plasmids, namely pSIH1-H1-Puro-miR-4433, pCMV-VSV-G and pCMV-△8.9, into 293T cells. After transfection for forty eight hours, viruses were collected by passing 45 μM filter, and added pre-seeded K562 cells. The positive K562-miR4433 cells were selected with puromycin and virus titer was calculated 48 hours post infection. With the virus titer, the number of K562 cells infected with the viruses can be estimated. The infected K562 cells then were subjected to cell viability assay, gene expression, luciferase activity assay, and tumor xenograft experiments.

### Nude mouse xenograft model

Male *nu/nu* BALB/c mice were bred at the animal facility of Kunming University of Science and Technology. The mice were housed in barrier facilities with a 12-h light dark cycle, with food and water available *ad libitum*. K562 cells (1× 10^7^) infected with miR-4433-carrying virus or control was inoculated subcutaneously on the flanks of 4- to 6- week-old nude mice. Tumors were measured every day with use of calipers. Tumor volumes were calculated using the following formula: *a^2^* × *b* × *0.4*, where *a* is the smallest diameter and *b* is the diameter perpendicular to *a*. The body weight, feeding behavior and motor activity of each animal were monitored as indicators of general health. The animals were then euthanized by cervical dislocation, and tumor xenografts were immediately removed, weighed and photographed. All animal studies were conducted with the approval of the Kunming University of Science and Technology Institutional Animal Care and Use Committee.

### Statistical analysis

GraphPad Prism 5.0 software (San Diego, CA) was used for statistical analysis. All experiments were performed at least thrice, and results were expressed as mean ± standard error (SE), unless otherwise stated. Comparisons between two groups used Student's *t* test, and comparisons among multiple groups involved one-way ANOVA with post-hoc intergroup comparisons using Tukey test. *P* < 0.05 was considered statistically significant.

## Results

### SAHA and SB down-regulated the Bcr-Abl mRNA level

To examine whether SAHA and SB affect the Bcr-Abl mRNA level, the CML cell lines K562, KBM5 and KBM5-T315I were treated with indicated concentrations of SAHA or SB for 48 hours (Fig. 1A and B), then Bcr-Abl mRNA levels of CML cells were evaluated using real-time qPCR. The results demonstrated that SAHA or SB potently inhibited the Bcr-Abl mRNA level in CML cell lines K562, KBM5 and KBM5-T315I, suggesting SAHA and SB down-regulated Bcr-Abl mRNA level at transcriptional or post-transcriptional levels.

### SAHA inhibited the growth of CML cells

**Figure 2:**
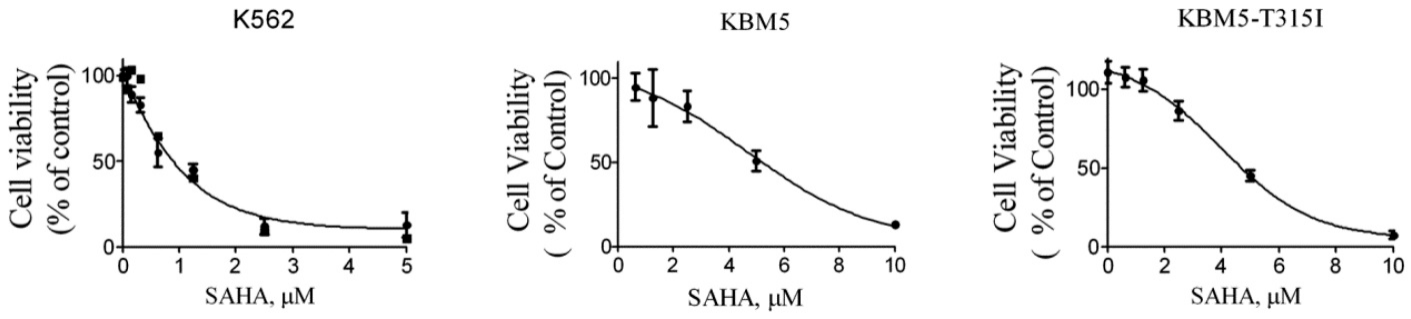
SAHA inhibited the growth of CML cell lines. Cell viability (percent relative to control) was determined by MTS assay. SAHA (72 hours treatment) potently inhibited the cell viability in K562, KBM5 and KBM5-T315I CML cells. Points, mean; bars, SE.

Next, in order to investigate the effects of SAHA on the growth of CML cells, the cell lines K562, KBM5 and KBM5-T315I were treated with increasing concentration of SAHA for 72 hours, and cell viabilities were evaluated using MTS assay. The cell viability of all cell types were effectively inhibited with IC_50_ values of 1.25 μM (K562), 4.66 μM (KBM5) and 5.7 μM (KBM5-T315I), respectively (Fig. 2). The data suggested that CML cell lines including the T315I mutant were sensitive to SAHA.

### SAHA decreased Bcr-Abl protein levels in CML cells

**Figure 3:**
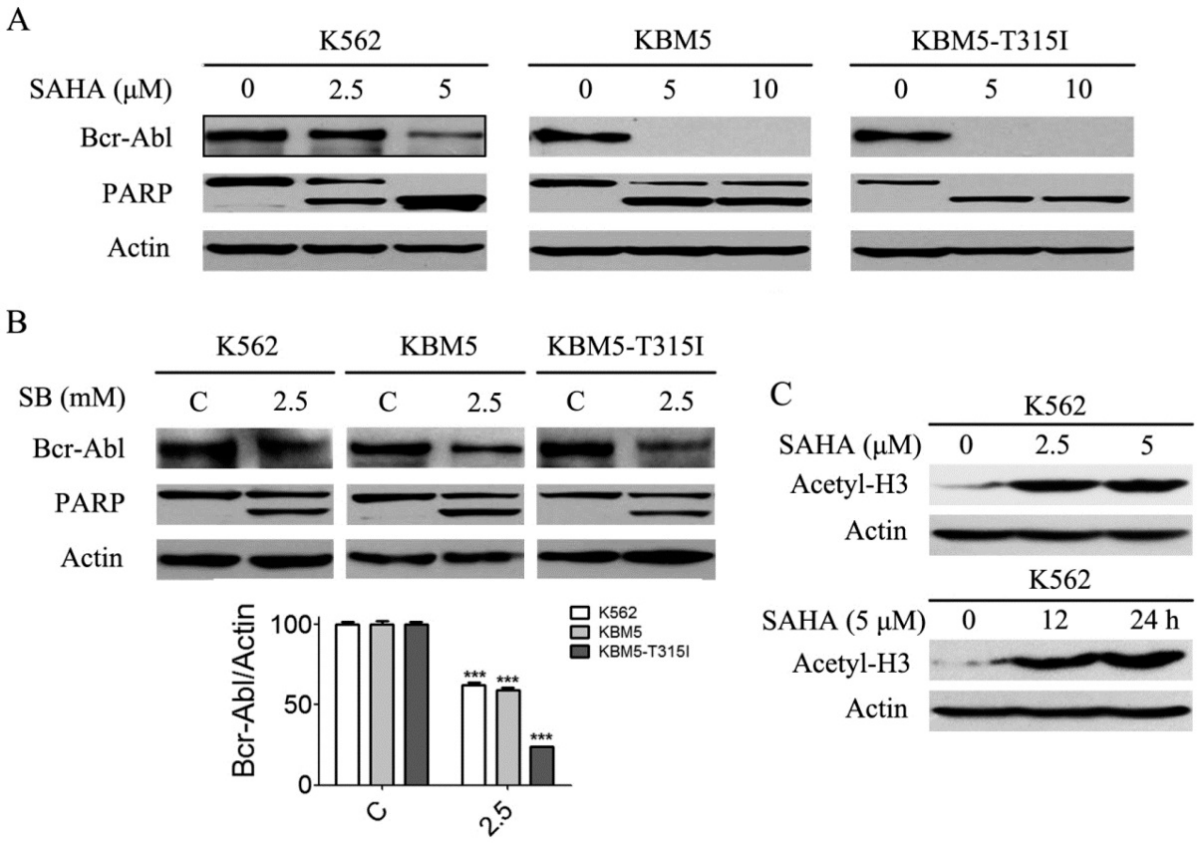
SAHA and SB decreased the Bcr-Abl protein levels and induced PARP specific cleavage, and SAHA up-regulated the histone acetylation level of H3 in K562 cells. (A) CML cells K562, KBM5 and KBM5-T315I were treated with 0, 2.5 and 5 μM SAHA for 48 hours, then the Bcr-Abl and PARP protein levels were examined by western blot analysis. (B) K562, KBM5 and KBM5-T315I cells were treated with 0 and 2.5 mM SB for 48 hours, and Bcr-Abl and PARP protein levels were examined by western blot analysis (upper). Meanwhile, Bcr-Abl protein density normalized to the internal control Actin (lower) by using Image-Pro Plus Version 6.0 software, Media Cybernetics, Inc. ***, *p* < 0.001, Student's *t* test. (C) K562 cells were treated with 0, 2.5 and 5 μM SAHA for 24 hours or treated with 5 μM SAHA for 12 and 24 hours, then acetylation level of H3 were determined by western blot analysis.

Previous results (Fig. 1A and B) revealed that SAHA and SB down-regulated Bcr-Abl mRNA levels in CML cells, and we presumed that Bcr-Abl protein levels will be affected. Thus, Bcr-Abl protein levels were analyzed using western blot. The results showed that SAHA and SB significantly diminished Bcr-Abl protein levels (Fig. 3A and B). Meanwhile, PARP, a hallmark protein of apoptosis, was specifically cleaved from 115 kDa to 85 KDa (Fig. 3A and B). Moreover, SAHA increased the protein level of acetyl-H3 in K562 cells (Fig. 3C).

### SAHA abrogated Bcr-Abl downstream signaling

**Figure 4:**
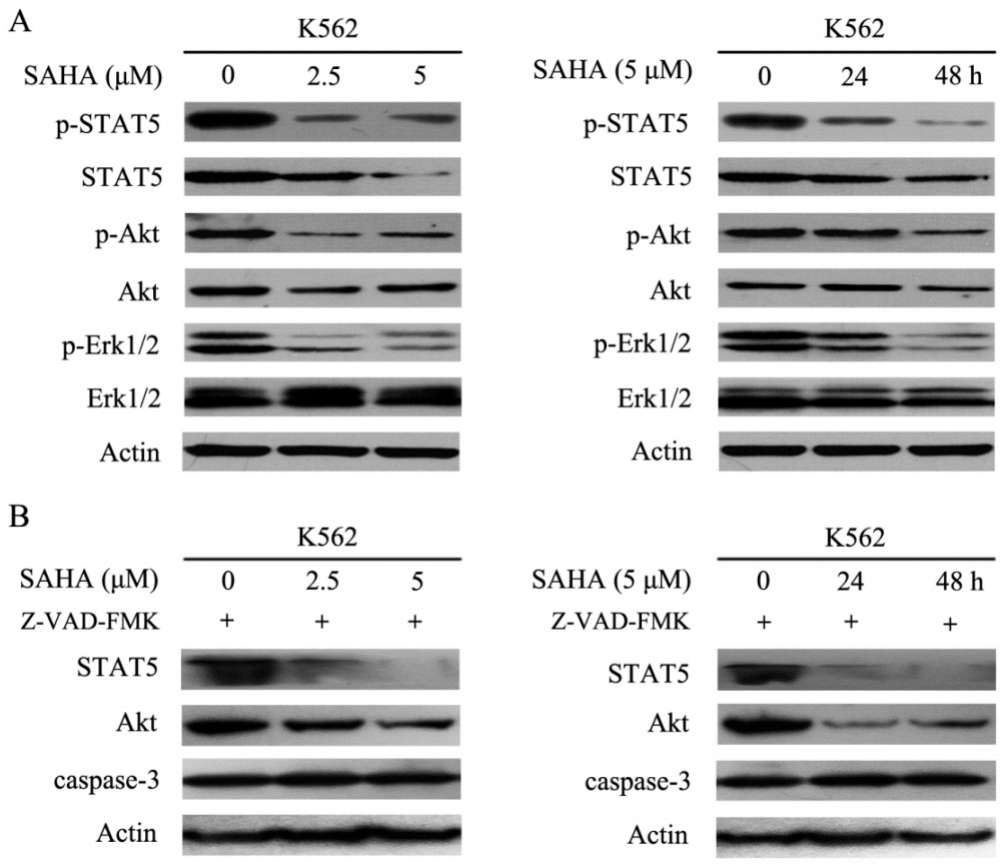
SAHA dose- and time- dependently abrogated the Bcr-Abl downstream signaling pathways. (A) K562 cells were exposed to SAHA with 0, 2.5 and 5 μM for 48 hours or 5μM for 24 and 48 hours, and then protein expression levels including p-STAT5, STAT5, p-Akt, Akt, p-Erk-1/2 and Erk-1/2 were analyzed by using western blot. (B) The SAHA treatment setting of K562 cells was the same as that of Fig. 4A except that 30 μM of Z-VAD-FMK treatment was added, followed by examination of the protein expression levels of STAT5, Akt and caspase-3.

Bcr-Abl harbors tyrosine kinase activity and can phosphorylate several downstream substrates and activate multiple signal transduction pathways such as STAT5, STAT3, PI3K/Akt, mitogen-activated protein kinase/extracellular signal-related protein kinase, and Crkl, all of which can stimulate cell proliferation and resistance to apoptosis 2. The data demonstrated that SAHA effectively inhibited phosphorylation of STAT5, Akt and Erk1/2, while reducing total Akt and STAT5 protein levels, but had no effect on total Erk1/2 protein levels in K562 cells (Fig. 4A). Additionally, SAHA-induced degradation of STAT5 and Akt could not be restored by Z-VAD-FMK which is a pan-caspases inhibitor (Fig. 4B). Therefore, SAHA decreased Bcr-Abl expression levels and further abrogated Bcr-Abl downstream signaling.

### SAHA increased the expression level of miR-4433

**Figure 5:**
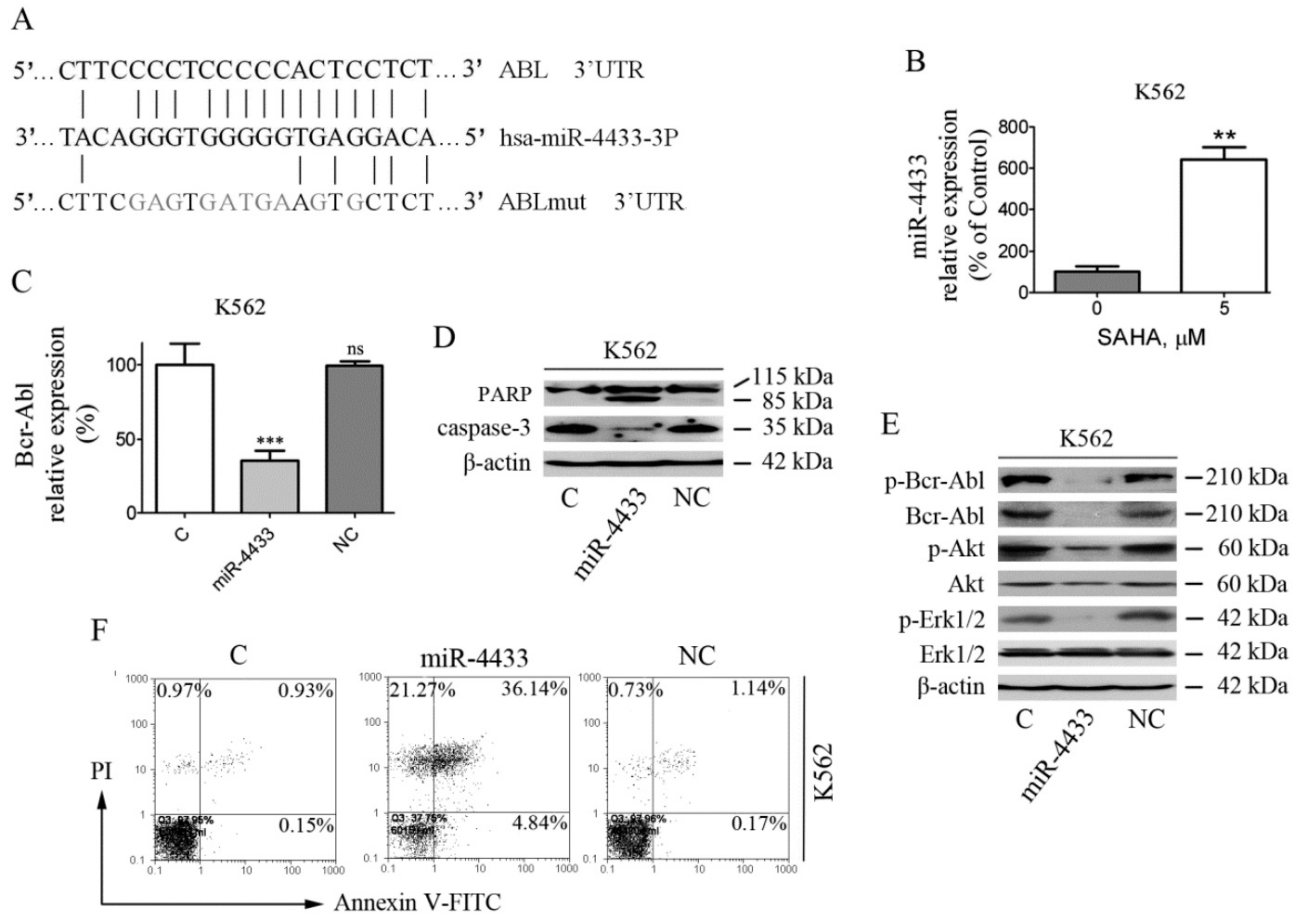
Transiently over-expressed miR-4433 inhibited the expression of Bcr-Abl and induced apoptosis in K562 cells. (A) Bioinformatics analysis of predicted miR-4433 binding sites on the 3'UTR of the ABL gene. A mutant ABL 3'UTR construct was prepared by mutating nucleotides in the seed region of the miR-4433 target site. (B) SAHA increased the expression level of miR-4433. K562 cells were exposed to SAHA (5 μM) for 48 hours and the miR-4433 expression level was determined by qRT-PCR. (C) K562 cells were transiently transfected with miR-4433, NC for negative control and C for control group, and then Bcr-Abl levels were determined by qRT-PCR. (D) Apoptosis-related proteins including PARP, caspase-3 were determined by western blot after the transfection in K562 cells. (E) p-Bcr-Abl, Bcr-Abl, p-Akt, Akt, p-Erk-1/2 and Erk-1/2 were examined by western blot post transfection in K562 cells. (F) Additionally, 48 hours post transfection of K562 cells, the apoptosis rates were measured by flow cytometry after Annexin V- FITC and propidium iodide (PI) double staining. Column, mean; bars, SE; **, *p* < 0.01, ***, *p* < 0.001, Student's *t* test.

Various reports demonstrated that microRNA expression was regulated epigenetically, such as methylation or acetylation regulation of microRNA gene expression 15, 16. We supposed that SAHA will up-regulate the expression of some microRNAs that target Bcr-Abl mRNA. Under this assumption, a series of microRNAs which supposed to target Bcr-Abl were selected using the online tools including TargetScan, miRBase, PicTac and miRanda. Then, the supposed Bcr-Abl-targeted microRNA expression levels (305 microRNAs) were monitored by qRT-PCR after K562 cells were treated with SAHA (5 μM). Thereafter, SAHA up-regulated microRNAs (21 microRNAs) were transfected into K562 respectively, and Bcr-Abl mRNA level was detected by qRT-PCR (data not shown). After microRNA expression screening, miR-4433-3p were identified since its expression level increased by 6.43-fold after SAHA treatment (Fig. 5B), and putative targeted seed sequence of miR-4433 on Bcr-Abl 3'UTR was showed in Fig. 5A, △G is -23.4 kcal/mol, which is the highest △G in the up-regulated microRNAs that may bind to Bcr-Abl 3'UTR.

### miR-4433 reduced Bcr-Abl and its downstream signaling

Furthermore, K562 cells were transfected with the synthetic mimics and then examined for Bcr-Abl and its downstream signaling. The results demonstrated that miR-4433 significantly reduced Bcr-Abl mRNA levels, however with no effect for control (C) and negative control (NC) groups on Bcr-Abl mRNA level (Fig. 5C). In addition, the phosphorylated Bcr-Abl, Bcr-Abl, phosphorylated Akt and phosphorylated Erk1/2 were inhibited after transfection, similarly with no effect for C and NC groups (Fig. 5E).

### miR-4433 induced K562 cells apoptosis

After K562 cells were transfected, cell apoptosis and apoptosis-related proteins were determined by flow cytometry and western blot, respectively. The results manifested that cell apoptosis was induced in K562 cells after transfection (Fig. 5F), accompanied by PARP specific cleavage and reduction of caspase-3 (Fig. 5D), whereas had no effect for C and NC groups.

### Bcr-Abl is a direct target of miR-4433

**Figure 6:**
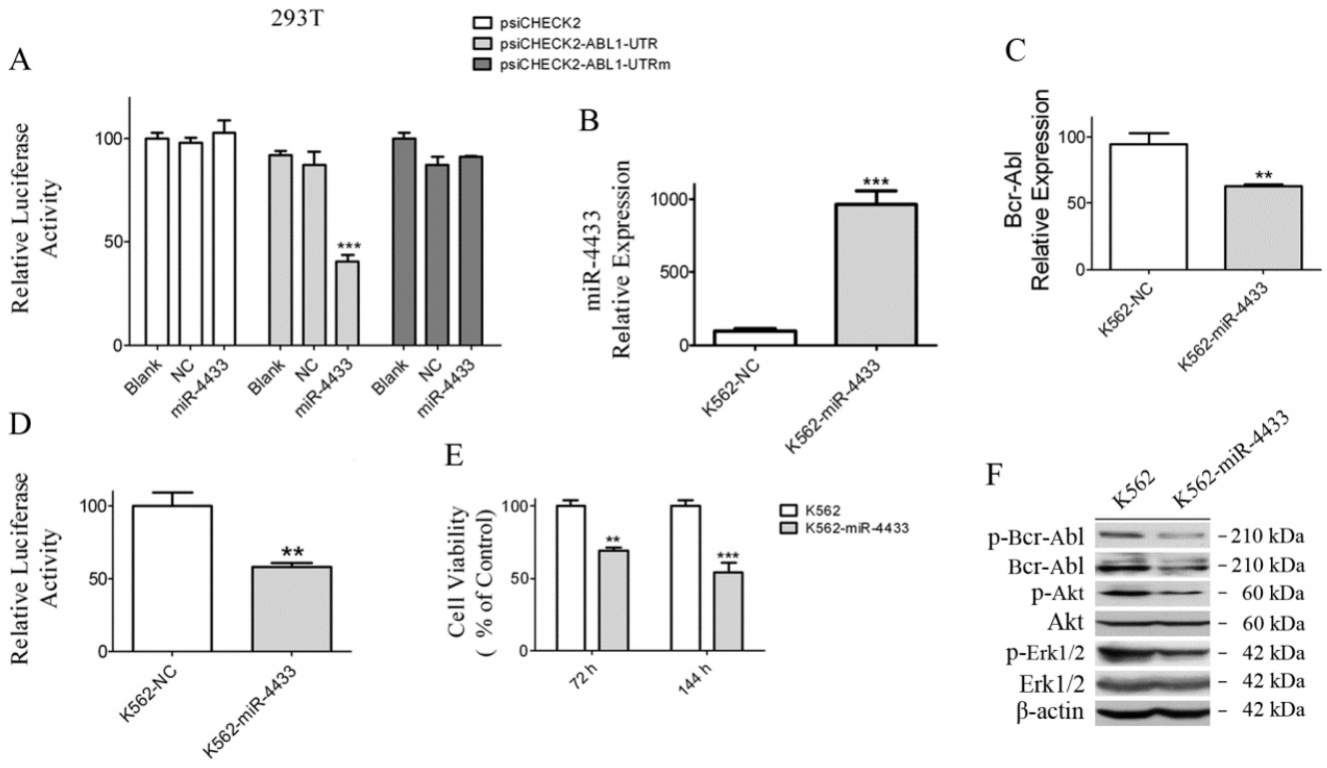
Characterization of miR-4433 as Bcr-Abl-targeted microRNA and over-expression of miR-4433 reduced Bcr-Abl expression level and inhibited K562 cells growth. (A) 293T cells were co-transfected with indicated plasmid (psiCHECK2 or psiCHECK2-ABL1-UTR or psiCHECK2-ABL1-UTRm) and microRNA mimics (miR-4433 or NC or Blank), and relative luciferase activity were determined. The miR-4433 (B) and Bcr-Abl (C) expression levels were measured by qRT-PCR in K562 cells stably expressing miR-4433 (K562-miR-4433). (D) Relative luciferase activity of K562-miR-4433 cells was determined after transfection with psiCHECK2-ABL1-UTR for 48 hours. (E) Cell viability of K562-miR4433 was determined after K562 cells were infected with the viruses carrying miR-4433. For the 144-hour infection group, at 72 hours, cells were equally divided into 8 wells using subculture, and cell viability was measured after further 72 hours of culture. (F) 72 hours post infection with miR-4433 carrying virus, the proteins including p-Bcr-Abl, Bcr-Abl, p-Akt, Akt, p-Erk-1/2 and Erk-1/2 were determined by western blot analysis. Column, mean; bars, SE; **, *p* < 0.01, ***, *p* < 0.001, Student's *t* test.

To further determine whether Bcr-Abl is a bona fide target of miR-4433, the 3'UTR of the human ABL1 gene was cloned into psiCHECK2, and reporter luciferase assay was performed. The relative luciferase activity of the reporter containing the wild-type ABL1 3'UTR was significantly inhibited when miR-4433 was co-transfected, whereas the relative luciferase activity of the mutant ABL1 3'UTR lacking the miR-4433 binding site was not affected (Fig. 6A), meanwhile there was no effect on luciferase activity of the Blank and the NC groups.

### miR-4433 expression abrogated Bcr-Abl and inhibited K562 cell growth

In order to examine whether K562 cells were affected when miR-4433 were stably expressed in K562 cells, an expression cassette for miR-4433 was cloned into the pSIH1-H1-Puro vector, thereafter the lentivirus carrying miR-4433 was packaged for infection of K562 cells. After infection for 48 hours, miR-4433 transcription level was quantified. The results revealed that the miR-4433 level was increased by 9.65-folds (Fig. 6B), indicating that miR-4433 was stably expressed in K562 cells. Secondly, expression of miR-4433 diminished the transcription level of Bcr-Abl in K562 cells (Fig. 6C). Furthermore, luciferase activity assay demonstrated that expression of miR-4433 reduced reporter luciferase activity when K562 cells were transfected with psiCHECK2-ABL1-UTR (Fig. 6D). Moreover, cell growth was significantly inhibited at 72 hours and 144 hours by sub-culture in K562 cells post infection with the virus carrying the miR-4433 (Fig. 6E). In addition, Bcr-Abl protein and its downstream signaling proteins were analyzed using western blot. The data indicated that phosphorylated Bcr-Abl, Bcr-Abl, phosphorylated Akt and phosphorylated Erk1/2 were reduced in K562 cells expressing miR-4433 (Fig. 6F).

### miR-4433 inhibited the growth of xenografted K562 cells in nude mice

**Figure 7:**
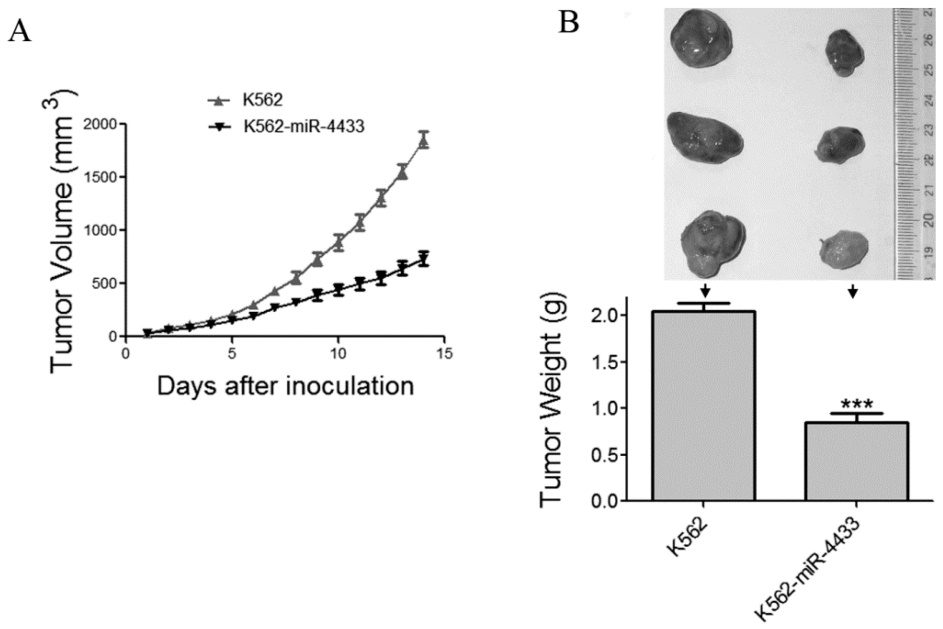
Expression of miR-4433 in K562 cells abrogated growth of tumors transplanted in nude mice. (A) The growth curves of subcutaneous xenografts of K562 and K562-miR-4433 cells are shown. The estimated tumor sizes are plotted against the number of days since tumor inoculation. (B) On day 14, xenografts were dissected, photographed, and weighed. Representative tumors removed from three mice of each group are shown (B, top). The bar chart (B, bottom) shows the weight of the tumors from each group (n = 12). Point, mean; bars, SE;

The in vitro anti-tumor effects of miR-4433 in K562 cells prompted us to examine the in vivo anti-tumor effects of miR-4433 using nude mouse xenografted model. Twelve *nu/nu* BALB/c mice were subcutaneously inoculated with K562 cells and another 12 mice with K562-miR4433 cells. The growth curves (the estimated tumor size calculated from the tumor dimension versus time) are shown in Fig. 7A. miR-4433 potently inhibited the growth of K562 cell tumors. The sizes of dissected tumors of K562-miR-4433 group were remarkably lower than that of K562 group (Fig. 7B, top). The weight of K562-miR-4433 tumors was significantly lower than that of K562 tumors (Fig. 7B, bottom). Taken together, these data demonstrated the anti-tumor effect of miR-4433 on K562 cells in vivo. The body weight of the mice remained stable and there was no significant difference between the two groups (data not shown). The motor activity and feeding behavior of the mice were all normal.

## Discussion

Chronic myelogenous leukemia is a myeloproliferative disease caused by the formation of the oncogenic Bcr-Abl, and targeting Bcr-Abl is the key to the treatment of CML. In this study, SAHA was used to screen for microRNAs targeting oncogenic Bcr-Abl. SAHA (vorinostat, Zolinza) is a patent 19, 20, reversible pan-histone deacetylase (HDAC) inhibitor, which has been approved by the FDA for treatment of cutaneous T-cell lymphoma 21. Our results indicated that SAHA potently increased the level of acetylated histone 3 and diminished Bcr-Abl level, while inhibiting CML cells growth and inducing apoptosis. microRNA gene expression is regulated by histone acetylation levels of related microRNA gene 16, 22, 23. To identify SAHA-induced microRNAs that can target Bcr-Abl, online bioinformatics algorithms, qRT-PCR and transfection of microRNAs were used, and miR-4433 was identified. The data showed that overexpression of miR-4433 reduced Bcr-Abl level in K562 cells and inhibited cell growth. However, whether SAHA-upregulated miR-4433 was through an epigenetic manner or through an indirect manner remained to be further investigated.

Down-regulation of Bcr-Abl by SAHA or other HDACI has been reported by some researchers. A study reported that SAHA enhanced the antitumor activity of imatinib in Bcr-Abl expressing cells by abrogating both Bcr-Abl mRNA and protein levels 24. Their data indicated that Bcr-Abl down-regulation was independent on cell apoptosis, as Bcr-Abl degradation could not be reversed by the pan-caspase inhibitor z-vad-fmk. Similarly, z-vad-fmk did not reverse the down-regulation of Bcr-Abl downstream proteins STAT5 and Akt in our study. Similar studies have demonstrated a synergistic interactions of HDACIs with bortezomib or BCR/ABL kinase inhibitor KW-2449 or PLK1 inhibitors (such as BI2536) in imatinib-sensitive and resistant Bcr/Abl^+^ cells 25-28. However, these reports didn't mention the specific molecular mechanism of HDACs-induced Bcr-Abl down-regulation. In another study by Rekha , they reported that treatment with pan-HDAC inhibitors or depletion of HDAC6 by siRNA inhibited chaperone function of hsp90, resulting in polyubiquitylation and proteasomal degradation of hsp90 client proteins, including Bcr-Abl 29.

To date, a number of studies have reported microRNAs targeting Bcr-Abl, including miR-203 15, miR-451 30, miR138 31, miR-29b 32, miR-30e 33, miR-30a 34, miR-23a 35, miR-424 36, and miR-320a 37. In these studies, microRNA expressions were all down-regulated in both patients with CML and CML cell lines. Transient overexpression of these microRNA mimics in CML cell lines reduced Bcr-Abl expression and inhibited the growth of CML cell lines. Interestingly, two microRNAs, miR-203 and miR-23a 15, 35, were epigenetically silenced in CML patients and CML cell lines, and treatment with epigenetic drug 5-aza-2'-deoxycytidine restored the microRNA expression and reduced the growth of CML cell lines. It is similar to the regulation pattern of miR-4433 by SAHA in this study, together with targeted effect of miR-4433 on Bcr-Abl, which were manifested in an epigenetically regulated manner. However, whether the expression pattern of the rest microRNAs targeting Bcr-Abl is regulated by epigenetic manner remains to be elucidated. In addition, the expression patterns of miR-4433 in patients with primary CML were not examined in this study due to a shortage of CML patient samples in our lab. Whether miR-4433 is silenced just like microRNAs in other reported CML patients will be further investigated if CML samples were available.

Taken together, we report SAHA, a FDA approved and clinically used drug for cancer therapy, up-regulated the expression of miR-4433, and further identified miR-4433 targeting Bcr-Abl, while inhibiting CML cell growth and inducing cell apoptosis. Although the anti-tumor effects of SAHA and other histone deacetylation inhibitors have been well documented 17, 20, 38, such as by inducing cell cycle arrest and apoptosis, this study revealed from another angle that SAHA abrogated oncogenic Bcr-Abl and inhibited growth of CML cells by up-regulated expression of miR-4433, which may provide a new molecular basis for application of SAHA in the treatment of patients with CML.

## Supplementary Material

Supplementary table.
