# Supplementary material for: Overexpression of miR-4433 by suberoylanilide hydroxamic acid suppresses growth of CML cells and induces apoptosis through targeting Bcr-Abl
Source: J Cancer. 2019 Sep 7;10(23):5671–80. doi: 10.7150/jca.34972 (PMC6843884; doi:10.7150/jca.34972)
Supplement: Supplementary file 1 — Supplementary table. [file jcav10p5671s1.pdf]

Table S1. Oligonucleotide sequences for PCR and overexpression of miR-4433

No.	nucleotide sequences	F/R
1	5’ CCCTCGAGCTGTCCACATCCCCAGAGCC 3’	F
2	5’ ATTTGCGGCCGCCCTAACCCTCCCTGATGACCC 3’	R
3	5’ AGAGCACTTCATCACTCGAAGGCCCTTTCAGGGCAC 3’	Rm
4	5’ CTTCGAGTGATGAAGTGCTCTAGACAAAGTAGATTCTTAC 3’	Fm
5	5’ CCCTCGAGCTGTCCACATCCC 3’	F
6	5’ ATTTGCGGCCGCCCTAACCCTC 3’	R
7	5’GATCCATGTCCCACCCCCACTCCTGTCTTCCTGTCAGAACAG GAGTGGGGGTGGGACATTTTTTG 3’	F
8	5'AATTCAAAAAATGTCCCACCCCCACTCCTGTTCTGACAGGAA GACAGGAGTGGGGGTGGGACATG 3’	R

1 and 2, primers for amplification of ABL1 3’UTR containing the miR-4433 targeted sequences; 3,4,5 and 6, primers for overlap-extension PCR to mutate the miR-4433 targeted sequences on ABL1 3’UTR; 7 and 8, oligonucleotide sequences for construction of miR-4433 overexpression plasmid; F, forward; R, reverse; m, mutation.
